# Estimation of total mediation effect for high-dimensional omics mediators

**DOI:** 10.1186/s12859-021-04322-1

**Published:** 2021-08-23

**Authors:** Tianzhong Yang, Jingbo Niu, Han Chen, Peng Wei

**Affiliations:** 1 Department of Biostatistics, The University of Texas MD Anderson Cancer Center, Houston, USA; 2 Department of Biostatistics and Data Science, School of Public Health, The University of Texas Health Science Center at Houston, Houston, USA; 3 Division of Biostatistics, University of Minnesota, Minneapolis, USA; 4 Section of Nephrology, Baylor College of Medicine, Houston, USA; 5 Human Genetics Center, Department of Epidemiology, Human Genetics and Environmental Sciences, School of Public Health, The University of Texas Health Science Center at Houston, Houston, USA; 6 Center for Precision Health, School of Public Health and School of Biomedical Informatics, The University of Texas Health Science Center at Houston, Houston, USA

**Keywords:** Aging, High-dimensional mediators, Iterative sure independence screening, Mediation analysis, $R^2$-based effect

## Abstract

**Background:**

Environmental exposures can regulate intermediate molecular phenotypes, such as gene expression, by different mechanisms and thereby lead to various health outcomes. It is of significant scientific interest to unravel the role of potentially high-dimensional intermediate phenotypes in the relationship between environmental exposure and traits. Mediation analysis is an important tool for investigating such relationships. However, it has mainly focused on low-dimensional settings, and there is a lack of a good measure of the total mediation effect. Here, we extend an R-squared (R$^2$) effect size measure, originally proposed in the single-mediator setting, to the moderate- and high-dimensional mediator settings in the mixed model framework.

**Results:**

Based on extensive simulations, we compare our measure and estimation procedure with several frequently used mediation measures, including product, proportion, and ratio measures. Our R$^2$-based second-moment measure has small bias and variance under the correctly specified model. To mitigate potential bias induced by non-mediators, we examine two variable selection procedures, i.e., iterative sure independence screening and false discovery rate control, to exclude the non-mediators. We establish the consistency of the proposed estimation procedures and introduce a resampling-based confidence interval. By applying the proposed estimation procedure, we found that 38% of the age-related variations in systolic blood pressure can be explained by gene expression profiles in the Framingham Heart Study of 1711 individuals. An R package “RsqMed” is available on CRAN.

**Conclusion:**

R-squared (R$^2$) is an effective and efficient measure for total mediation effect especially under high-dimensional setting.

**Supplementary Information:**

The online version contains supplementary material available at 10.1186/s12859-021-04322-1.

## Background

Understanding the relationships between an environmental risk factor and health traits through molecular phenotypes, such as gene expression (GE) and DNA methylation, can provide mechanistic insights into disease etiology and exposure biology. Specifically, an environmental risk factor may lead to epigenetic changes, such as changes in DNA methylation, which then alter DNA accessibility and chromatin structure, and thereby regulate GE and further downstream molecular phenotypes pertinent to the disease process. Modern epidemiological studies are capable of measuring a large number of markers, from tens of thousands of GEs to nearly a million CpG sites in DNA methylation studies. There is growing evidence that many of these intermediate phenotypes could lie in the pathway between environmental exposure and downstream health outcomes [1, 2]. It is of great scientific interest regarding how to measure the overall contribution of different types of molecular phenotypes in the pathways from an environmental risk factor to a phenotype endpoint. Mediation analysis is a natural approach to explore such relationships, which can help researchers delineate why and how two variables (dependent variable and independent variable) are related [3].

Our motivating scientific question here is how chronological age affects different health traits through molecular phenotypes. Specifically, we are interested in exploring the mediating role of GEs in the pathway between age and two health traits, blood pressure (BP) and lung function. As an important risk factor for a wide range of health conditions, age can be regarded as a proxy of lifestyle, oxidative stress, or other accumulated environmental risk factors. Researchers have found that GE profiles are associated with the aging process in various biological pathways, notably those involving overexpression of inflammation and immune response genes and underexpression of collagen and energy metabolism genes [4, 5]. On the other hand, a decrease in lung function and increase in systolic BP were found to be associated with many age-related changes, including inflammation and altered immunity, and these changes may be reflected on the molecular level [6–9]. Instead of exploring the mediating effect of a particular gene, we intend to quantify the overall role of potentially high-dimensional GEs in mediating the relationship between age and health traits, i.e., the total mediation effect. To the best of our knowledge, the existing total mediation effect size measures have been studied under low-dimensional settings and many of them are based on the difference in means, i.e., first-moment estimand (to be detailed later). Less attention has been given to the moderate- and high-dimensional settings [10], although such a measure may be especially useful in guiding further more specific analyses and providing mechanistic insights.

To fill in the gap, we extend a total mediation effect size measure, the R-squared (R$^2$) measure, which was originally proposed in a single-mediator model by Fairchild et al. [11], to the multiple- and high-dimensional mediator models. Briefly, the R$^2$ measure is a second-moment measure, quantifying the amount of variance in the dependent variable that is common to both the independent variable and the mediator(s), derived from commonality analysis [12, 13]. As an estimand based on variation, it provides an alternative to existing measures, especially in the presence of possible opposite directions of mediation effects as reported in the literature [14, 15] and our motivating example (Additional file 1: Fig. S3). We show that the R$^2$-based second-moment measure has many statistical merits and is easy to interpret. Additionally, our estimation method based on mixed-effect models can accommodate multiple and high-dimensional mediators well. However, when addressing our motivating question in the real data, we face an additional challenge that the identification of the true mediators is not known a priori. This is, in fact, not trivial for any similar questions with high dimensionality. We establish a consistent estimation procedure that first uses a variable selection method with the oracle property [16] to filter out the non-mediators that bias the R$^2$-based second-moment measure, and then obtains stable R$^2$ estimates based on the selected mediators. In addition to theoretical justification, we conduct extensive simulations from various perspectives, including bias, variance, finite sample performance of consistency, and the coverage probability of the confidence interval (CI). We show that our method has an all-around performance. We then apply it to answer our motivating question using the Framingham Heart Study (FHS) data, which contains a total of 17,873 candidate genes with corresponding GEs, 1711 subjects for BP evaluation, and 1378 subjects for lung function evaluation. Since the GE levels in the FHS were measured at the same time, we assume undirected correlation among the GE levels, following Huang and Pan 2016 [17] and Boca et al 2013 [18]. Nonetheless, we demonstrate that the R$^2$-based second-moment measure is also viable to use when there are directed paths among mediators, i.e., mediators are conditionally dependent on the exposure. The main consideration of our study is the magnitude of the total mediation effect, instead of hypothesis testing that considers whether the effect is present or not [17–20].

## Results

### Simulation results

#### Simulation setting I

Table 1 presents the bias and variance under the high-dimensional settings, i.e., (H1) to (H5) as detailed in Methods. When the model consisted of the true mediators (H1, H5), non-mediators $\mathbf{M} ^{(1)}$ (H3), and noise variables (H4), the $R^2_{Mediated}$ estimators had very small bias and variance. Estimators of the product, proportion, and ratio measures had relatively high bias when $n=p_0$ under scenarios (H2) to (H4), probably because it required estimating a large number of coefficients. In addition, the $R^2_{Mediated}$ estimators were biased under scenario (H2) as expected, suggesting the importance of excluding non-mediators $\mathbf{M} ^{(2)}$. We further confirmed that our normal assumption on the distribution of random effects was quite robust to misspecification (scenarios (H6)-(H12) as discussed in Additional file 1: Section 1.5.3 and shown in Table S3). On the other hand, under low-dimensional setting, we found that mixed-effect models had a slightly better performance in estimating $R^2_{Mediated}$ and the shared over simple effect (SOS) as defined in Methods, compared with fixed-effect models; however, fixed-effect models had a better performance in estimating the product, proportion, and ratio measure (Additional file 1: Table S1).

**Table 1:** Bias and standard deviation under high-dimensional settings (Simulation setting I): bias in the first row, and standard deviation in the second row for each scenario

	$R^2_{Mediated}$	SOS	ab	ab (Lasso)	prop	prop (Lasso)	ratio	ratio (Lasso)
H1	0.0006	0.0013	$-0.0084$	$-0.0324$	0.0001	0.0069	$-0.0107$	$-0.0231$
($\hat{{\mathbf {M}}} = \mathbf{M }$)	(0.0181)	(0.0370)	(0.2846)	(0.2744)	(0.0833)	(0.0795)	(0.1161)	(0.1117)
H2	0.0146	0.0299	0.1602	$-0.0359$	$-0.0493$	0.0058	0.0075	$-0.0212$
($\hat{{\mathbf {M}}} = [\mathbf{M }, \mathbf{M }^{{(2)}}]$)	(0.0184)	(0.0375)	(0.6463)	(0.2604)	(0.1960)	(0.0777)	(0.2886)	(0.1165)
H3	0.0006	0.0053	0.0923	0.0547	$-0.0552$	$-0.0520$	$-0.0013$	$-0.0315$
($\hat{{\mathbf {M}}} = [\mathbf{M }, \mathbf{M }^{{(1)}}]$)	(0.0071)	(0.0653)	(0.7443)	(0.7547)	(0.2520)	(0.2495)	(0.2983)	(0.3392)
H4	0.0047	0.0095	0.1421	$-0.0347$	$-0.0447$	0.0025	0.0498	$-0.0196$
($\hat{{\mathbf {M}}} = [\mathbf{M }, noise]$)	(0.0198)	(0.0403)	(0.2613)	(0.2519)	(0.0785)	(0.0689)	(0.0982)	(0.1055)
H5	$-0.0000$	$-0.0000$	$-0.0867$	$-0.3449$	$-0.0173$	$-0.0293$	$-0.0532$	$-0.2327$
($\hat{{\mathbf {M}}} = \mathbf{M }$)	(0.0095)	(0.0109)	(0.0956)	(0.1618)	(0.0158)	(0.0184)	(0.0482)	(0.0295)

ab: product measure; prop: proportion measure. (Lasso) indicates that the estimation is based on the Lasso regression; otherwise, it is estimated by a mixed-effect model. The true values are presented in Additional file 1: Table S2. The set of variables included in the model is denoted as $\hat{{\mathbf {M}}}$. The set of true mediators is denoted as $\mathbf{M}$, the set of variables associated with exposure but not with outcome is denoted as $\mathbf {M}^{\mathbf {(1)}}$, and the set of variables associated with outcome but not the exposure is denoted as $\mathbf {M^{(2)}}$. Variables in $\mathbf {M}^{\mathbf {(1)}}$ and $\mathbf {M^{(2)}}$ are non-mediators falsely included in the putative mediator set $\hat{{\mathbf {M}}}$

#### Simulation setting II

**Fig. 1:**
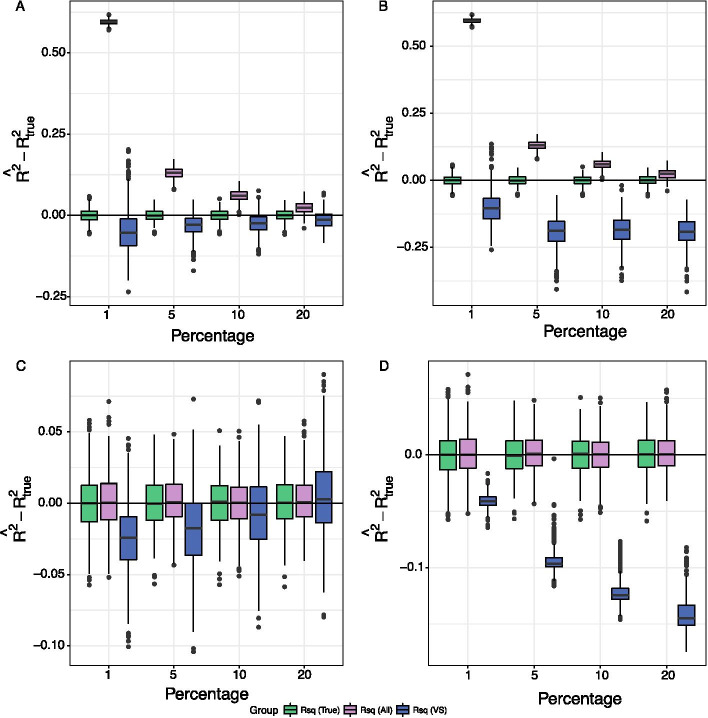
Boxplots of the bias across simulation replications based on a two-step variable selection method, either the iterative SIS or FDR (simulation setting II). X-axis corresponds to the percentage of true mediators; Y-axis corresponds bias across simulation replications. **A**, **B** non-mediators $\mathbf{M} ^{(2)}$ are included in addition to the true mediators; **C**, **D** non-mediators $\mathbf{M} ^{(1)}$ are included in addition to the true mediators. Rsq (All):$R^2_{Mediated}$ based on all the data without variable selection; Rsq(VS): $R^2_{Mediated}$ based on the variables selected either by iterative SIS (**A**, **C**), or by FDR (**B**, **D**); Rsq (True): $R^2_{Mediated}$ based on the true model/mediator set based on all the data. The numerical values and the bias and variance corresponding to the none mediators (null model) are available in Additional file 1: Tables S4 and S5

**Fig. 2:**
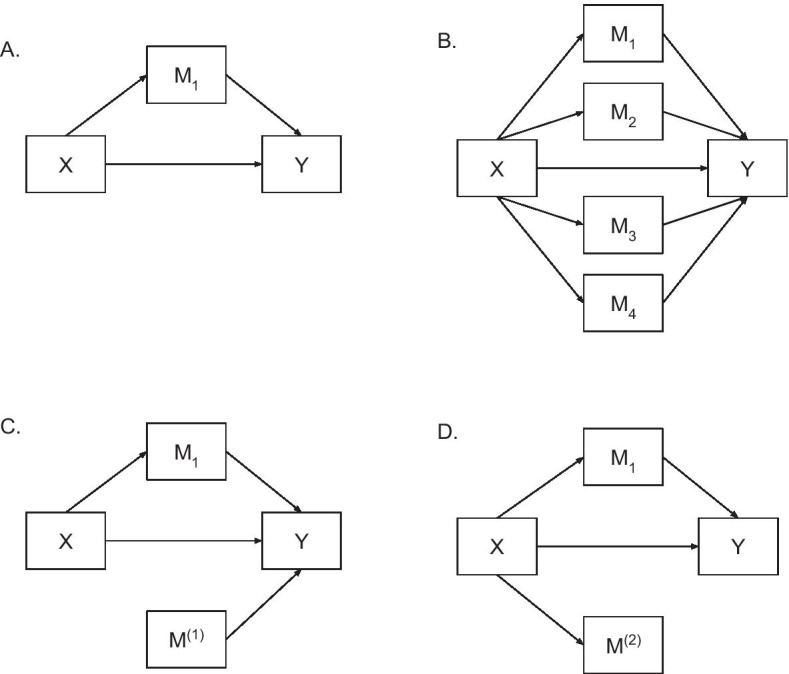
Demonstration of mediation analysis. X is the independent variable, Y is the dependent variable, and $M_j$ is the true mediator; **A** Single-mediator model; **B** Multiple-mediator model; **C** shows $M^{(1)}$ that is a non-mediator not associated with X, but with Y; **D** demonstrates a non-mediator $M^{(2)}$ that is associated with X, but not associated with Y after adjusting for X

We examined the performance of using iterative sure independence screening (SIS) and false discovery rate (FDR) to select the true mediators ${\mathbf {M}}$ from $\mathbf {M}_\mathbf{0 }$. Figure 1 shows the bias of $R^2_{Mediated}$ using iterative SIS and FDR to perform variable selection when $\mathbf{M} ^{(2)}$ or $\mathbf{M} ^{(1)}$ were included. The numerical values of the bias, SD, and MSE of the $R^2_{Mediated}$ and the product measure estimated by Lasso regression are presented in Additional file 1: Tables S6 and S7. We found that: (1) when only $\mathbf{M} ^{(1)}$ existed, using an inappropriate variable selection method, i.e., FDR, introduced large bias (Fig. 1D); (2) when $\mathbf{M} ^{(2)}$ existed, applying iterative SIS reduced bias to a much smaller scale, while including all variables without variable selection had a large amount of bias (Fig.1A). The FDR method was so conservative in picking up the true mediators, i.e., low true positive rates, that the bias was changed to negative values (Fig. 1B, D). Although not shown, we varied the FDR cutoffs from 0.01 to 0.25 and found that a more liberal cutoff sometimes better controlled the amount of bias, depending on the percentage of true mediators. Nonetheless, the true proportion of mediators is usually unknown. Therefore, we decided to use iterative SIS for variable selection in the following analyses. The results did not change much in terms of bias, standard deviation (SD), and mean square of error (MSE) with a much larger number of putative mediators, i.e. $p_0=15,000$ (see Additional file 1: Section 1.6.1 for the details).

#### Simulation setting III

We further evaluated the finite-sample performance of the iterative SIS variable selection coupled with the mixed-effect estimation procedure for $R^2_{Mediated}$ . As sample size increased, the bias and SD of ${\hat{R}}^2_{Mediated}$ decreased, with a more precise selection of the true mediators (average true positive rates and false positive rates are reported in Additional file 1: Table S6). In addition, we evaluated the coverage probability of the bootstrap-based CI at different numbers of true mediators with a sample size of 1500. We found that when the number of true mediators was 0, and, therefore, the true $R^2_{Mediated}$ was 0, none of the mediators was selected in all bootstrap samples across simulation replications, leading to a constant 0 estimate. Moreover, 98.0%, 98.0%, and 94.5% of the CIs covered the true value when the number of true mediators was 15, 150, and 300, respectively. Lastly, we did observe a worse performance in variable selection when the mediators were highly correlated with a given sample size, although the bias and variance of the $R^2_{Mediated}$ did not deteriorate too much (Additional file 1: Table (S7)). We also observed that regressing out the covariates as proposed in Additional file 1: Section 1.4 could help improve the performance of variable selection by reducing the correlations among mediators due to potential exposure-mediator confounders (Additional file 1: Section 1.7.4 and Table S8).

### Real data example: the Framingham Heart Study

We hypothesized that the effect of chronological age on lung function or systolic BP was mediated by changes in GE levels. We performed a mediation analysis on the FHS Offspring Cohort of European ancestry who attended the eighth and ninth examinations with the average interval between visits being around 6 years. Lung function was measured by forced vital capacity (FVC) in liters, using the highest value among at least two acceptable maneuvers. BP was measured as an average of two sequential readings in mmHg. 15 mmHg was added to the systolic BP if a participant reported taking antihypertensive medication at the time of BP measurement [21]. The covariates were the demographic variables of weight in lb, sex, height in inches and smoking status (ever vs never). We focused on subjects with non-missing measurements on the covariates variables, phenotype of interest, and pedigree information, resulting in a final sample size of 1378 for FVC and 1711 for systolic BP. We tackled the inter-individual correlation in phenotypes, due to family relatedness, by taking residuals of a linear mixed model with a random effect following a multivariate normal distribution with a zero mean vector and a covariance matrix proportional to the kinship matrix derived from the pedigree information [22]. GE profiling for 17,873 genes was measured from fasting peripheral whole blood using the Affymetrix Human Exon 1.0 ST GeneChip platform, details of which were described in previous publications [23]. We used age and GE levels at the eighth examination, and FVC and systolic BP at the ninth examination, such that the temporal precedence from exposure to mediators and mediators to phenotype were established. To take into account the possible confounding effects, we regressed covariates out from age, pedigree relatedness-adjusted phenotypes, and 17,873 gene expression levels and used the resulting residuals in subsequent analyses (also see Additional file 1: Section 1.4 for a general estimation procedure involving covariates).

We assumed that a small proportion of genes were involved in the pathway from chronological age to the two health traits. As supported by our simulation study (Fig. 1), we did not conduct any pre-screening on $\mathbf{M} ^{(1)}$; instead, we only performed variable selection to exclude $\mathbf{M} ^{(2)}$. The results are summarized in Table 2. We found that the variance in FVC shared by chronological age and GE was estimated to be 0, whereas there was considerable shared variance in systolic BP. Specifically, after taking into account weight, height, sex, and smoking status as covariates, 20.7% of FVC variation could be explained by age, but the number of selected mediators using iterative SIS-MCP was 0 for FVC, suggesting that changes in GE levels did not impact FVC after adjusting for age. This was further confirmed using the Lasso regression and FDR control method. Since GE levels were collected from whole blood, rather than lung tissue, the GE levels in blood might be less relevant for lung function than for blood traits. On the other hand, we found that 6.9% of systolic BP variation can be explained by age, and 2.6% (95% CI = (− 0.3%,6.6%)) could be commonly explained by age given the covariates, and 182 genes whose GEs selected by iterative SIS, accounting for 38.1% (95% CI = (− 8.5%,77.1%)) of the variance explained by age, as measured by SOS. Note that based on the proportion measure, 0.8% (95% CI = (− 17%,14%)) of the total effect was mediated by GEs. Additionally, the CIs of ratio and product measure were almost symmetric around 0, suggesting the existence of bidirectional mediation effects from individual pathways. Additional file 1: Figure S3 also confirmed such relationships for both health traits.

**Table 2:** Mediation effect size estimated using the Framingham Heart Study data.

Outcome	*n*	$${{\hat{p}}}$$	$$R^2_{Y,X}$$	$$R^2_{Mediated}$$	SOS	ab$$^{1}$$	prop	ratio	$$\nu $$$$^{1}$$
FVC$^{2}$	1378	0	0.207	0	0	0	0	0	0
		(0, 0)	(0.153, 0.265)	(0, 0)	(0, 0)	(0, 0)	(0, 0)	(0, 0)	(0, 0)
Systolic BP	1711	207	0.069	0.026	0.381	0.002	0.008	0.008	4.1e−6
		(146, 224)	(0.035, 0.111)	(− 0.003, 0.066)	(− 0.085, 0.771)	(− 0.04, 0.03)	(− 0.17, 0.14)	(− 0.14, 0.16)	(1.1e−6, 1.8e−3)

95% CI is within the parentheses based on percentiles of 500 bootstrap samples; ${{\hat{p}}}$ is the number of genes in estimation; *n* is the sample size for each trait; A mixed model is used to estimate the quantities, including $R^2$’s, ab (the product measure), prop (the proportion measure), ratio, and the $\nu$ measure for multiple mediators

^1^ab and $\nu$ were calculated based on standardized residuals with SD = 1

^2^Lasso and FDR methods were also applied on FVC, by which none of the gene was selected

We further conducted a pathway enrichment analysis of the selected mediators for systolic BP and four nominally significant pathways had biological evidence supporting their potential mediation role between age and systolic BP (Additional file 1: Table S9). For example, the nucleotide excision repair pathway was shown to be involved in age-related vascular dysfunction, which in turn is associated with hypertension [24]. Future analyses with larger sample sizes and using more relevant tissues are warranted to estimate the total mediation effects.

## Discussion

We have extended the existing R$^2$ measure, originally proposed in the single-mediator model, to multiple- and high-dimensional mediator models, for the purpose of applying this measure to high-dimensional omics mediators. Different from the estimation method of the single-mediator model, we proposed a top-down approach: instead of estimating every single regression coefficient, we estimated $R^2_{Mediated}$ based on the variance components of random coefficients in the mixed model framework. This method can be very efficient, particularly for huge numbers of mediators, because it greatly reduces the number of parameters needed to be estimated. The $R^2_{Mediated}$ is satisfactorily estimated with correctly-specified models, but identifying the true mediators under high-dimensional settings is a challenging problem. The $R^2_{Mediated}$ is biased when variables associated with the exposure, yet not with the dependent variable, are included. To this end, we showed that using iterative SIS can largely mitigate such bias, while using all available GEs led to overestimation, and using a hypothesis testing method with stringent FDR cutoff led to underestimation. To draw valid post-selection inference following the variable selection step, we split the data into halves: we use the first half for variable selection and the second half for estimation. But it is also possible and probably more efficient, though not yet thoroughly studied for iterative SIS, to use all the data (with certain adjustments) in a more unified framework [25]. We used the nonparametric bootstrap method to calculate the CI and showed that it has satisfactory coverage probability with the sample size comparable to the FHS data. We used the residuals of exposure, mediators and outcomes orthogonalized with respect to the covariates in the real data analysis. It helped improve the performance of variable selection compared with directly adjusting the covariates as shown in simulations (Additional file 1: Section 1.7.4). Additionally, it can be easily shown that the corresponding $R^2$’s are partial $R^2$, thus $R^2_{Mediated}$ is the additional amount of variance explained given the covariates (Additional file 1: Section 1.4.1).

$R^2_{Mediated}$ is an extremely useful measure because it can be objectively evaluated and compared across studies [26]. For example, we were able to compare the total mediation effects of the same exposure-trait pair through different types of molecular phenotypes, such as GE and DNA methylation [27], or GE in different tissues. We can also compare the total mediation effects of the same exposure and multiple traits through the same set of mediators. Using the FHS data set as our motivating example, we estimated $R^2_{Mediated}$ as a total mediation effect measure for age and two traits, i.e., FVC and systolic BP, by using the same set of GEs as candidate mediators. Age is an intriguing and important environmental exposure. Some studies used the methylation to predict biological age, which can serve as a proxy for overall health condition [28, 29]. We examined the relationship from a different perspective using mediation analysis. Interestingly, we found a large amount of age-related variation in systolic BP can be explained by GEs, while the product/proportion/ratio measures’ $95\%$ CIs were centered around 0 due to the bidirectional mediation effects from individual pathways.

Mediation analysis of molecular data can be prone to confounding and reverse causation [30]. It is of our future interest to develop the $R^2_{Mediated}$ measure under the longitudinal setting. Longitudinal analysis allows the examination of whether changes in GE profiles are more likely to precede changes in health traits. It can also deal with unmeasured confounding because each subject serves as a control for oneself.

$R^2_{Mediated}$ was previously considered to have only a heuristic value, mainly because it can be negative under certain circumstances. When that happens, researchers may find it difficult to interpret. We emphasize that the $R^2_{Mediated}$ measure is a second-order common effect and thus no longer a proportion measure [12]. To facilitate the use of $R^2_{Mediated}$, we evaluated the range of the $R^2_{Mediated}$ in Additional file 1: Section 1.2.3 Propositions 1–3. Generally, when the magnitude of the ratio of direct effect and total effect exceeds a certain threshold (larger than 1), $R^2_{Mediated}$ becomes negative; however, under high-dimensional settings, the threshold can be very high, such that the occurrence of negative value is infrequent. Finally, we have developed an R package ‘RsqMed’, which is publicly available on CRAN, to implement the proposed $R^2_{Mediated}$ measure estimation and its CI. The current development of the R$^2$-based second-moment measure is focused on continuous outcomes and only additive mediation effects without exposure-mediator interactions. Extensions to binary and time-to-event outcomes and non-additive mediation effects warrant further investigation.

## Conclusions

We presented a top-down approach for high-dimensional mediation analysis to answer our motivating question: how does gene expression mediate in the pathway between age and a health trait of interest. In FHS, we showed that gene expression played an important role in mediating the pathway from age to systolic blood pressure and interestingly, the selected mediators were enriched in the pathways related to inflammatory and age-related vascular dysfunction. The R$^2$ measure coupled with our proposed estimation method is generalizable and has many appealing statistical properties, such as its close connection with the existing measures, adaptivity to a complex dependent structure among mediations, having low bias and variance, consistent, and satisfactory coverage probability of confidence interval. In the multiple- and high-dimensional mediator model, it can serve as a good starting point to guide more specific downstream biological analyses.

## Methods

### Review of the commonly-used total effect size measures

A mediation model (Fig. 2) consists of the following equations. Without loss of generality, we assume the dependent, independent and mediator variables are standardized to have mean 0 and variance 1.

1 $$\begin{aligned}&Y=cX+e_1, \end{aligned}$$

2 $$\begin{aligned}&Y=r X+\sum _{j=1}^p M_jb_j+e_2, \end{aligned}$$

3 $$\begin{aligned}&M_j=a_jX+\xi _j. \end{aligned}$$

*p* is the total number of mediators. When $p=1$, it corresponds to a single-mediator model (Fig. 2A); otherwise, it corresponds to a multiple-mediator model (Fig. 2B). *Y* is the continuous dependent variable; *X* is the independent variable; $M_j$ is the *j* *th* mediator; $e_1,e_2$, and $\xi _j$ are residuals for each equation; $a_j,b_j,r$ and *c* are regression coefficients, usually estimated by the maximum likelihood estimation (MLE) method. Parameter *c* is the total effect and *r* is the direct effect.

Product, proportion, and ratio measures, all based on the difference in means, are among the most commonly seen total mediation effect measures in the literature. The product measure is $\sum _{j=1}^p a_jb_j$. It is also the natural indirect effect under the potential outcome framework with strong causal inference and model assumptions [31]. The proportion measure is defined as the proportion of total effect mediated by *M*: $\sum _{j=1}^p a_jb_j/(\sum _j^p a_jb_j+r)$; the ratio measure is $\sum _{j=1}^p a_jb_j/r.$ All three measures are sensitive to the direction of effects through different individual mediation pathways. In an extreme example, $a_jb_j$ from individual pathways have different directions and thus cancel out, result in sum of 0. It leads to a misleading implication that there is no mediation effect at all. Additionally, both the proportion and ratio measures are unit-free, but require a sample size larger than 500 to obtain stable estimates even under low-dimensional settings [3].

Another total mediation effect measure recently proposed by Song et al. [15] is $\sum _{j=1}^p (a_jb_j)^2$. As a quantity based on the L-2 norm, it overcomes the drawbacks mentioned above; however, it is less interpretable than the above three first-moment measures and the R$^2$-based second-moment measure to be described.

### R$^2$ measure under a single-mediator model

Compared with the aforementioned total mediation effect size measures, the R$^2$ measure has not drawn much attention. The R$^2$ measure is defined as the variance in dependent variable Y explained by the independent variable X through the mediator [11] (See the Venn diagram in Additional file 1: Fig. S1). It can be written as

$$\begin{aligned} R^2_{Mediated}=r^2_{Y,M}+r^2_{Y,X}-R^2_{Y,MX}, \end{aligned}$$

where $r^2$ in lower case denotes the variance explained in the simple regression model and is equal to the squared correlation coefficient; capital $R^2$ denotes the coefficient of determination for a multiple regression model. $r_{Y,M}^2=cor(Y,M_1)^2$ is the variance in Y explained by $M_1$ in the following model (4), $r_{Y,X}^2=cor(Y,X)^2$ is the variance in Y explained by X in model (1), and $R^2_{Y,MX}$ is the variance in Y explained by $M_1$ and X in model (2) with $p=1$.

4 $$\begin{aligned} Y= M_1d_1 +e_4, \end{aligned}$$

where $d_1$ is the regression coefficient and $e_4$ is the residual.

The three components in $R^2_{Mediated}$ can be estimated by the MLE using fixed-effect models, i.e., treating all the coefficients as fixed. We note that the $R^2_{Mediated}$ is a difference-in-R$^2$ measure, instead of a proportion measure. The R$^2$ measure has been recognized to have many characteristics of a good measure of effect size. For example, it has a stable performance for sample sizes $>100$ [11], it increases as the mediation effect approaches the total effect, and it is possible to construct a CI estimate. There are a few other variants of R$^2$ measure in the literature, such as those proposed in [3, 32] under a single-mediator model. They were aimed at different additional potential advantages including a bounded range between 0 and 1, a monotonic relationship with the product measure, and better dealing with spurious correlations, at the possible price of losing connection with the commonality analysis. More discussion is included in Additional file 1: Section 1.2.

### Extension: $R^2_{Mediated}$ under the multiple-mediator model

We extend the R$^2$ measure to the multiple-mediator model, defined as:

5 $$\begin{aligned} R^2_{Mediated}&= R^2_{Y,M}+r^2_{Y,X}-R^2_{Y,MX} \nonumber \\&=r^2_{Y,X} - (R^2_{Y,MX}-R^2_{Y,M}), \end{aligned}$$

where $r_{Y,X}=cor(Y,X),$ $R^2_{Y,MX}=var(rX + \sum _{j=1}^{p} M_jb_j),$ and $R^2_{Y,M} = var(\sum _{j=1}^{p} M_jd_j).$ $R^2_{Y,M}$, $r^2_{Y,X}$, and $R^2_{Y,MX}$ have the same meaning as in the single mediator models and the corresponding models are (6), (1) and (2) with $p>1$.

6 $$\begin{aligned} Y= \sum _{j=1}^{p} M_jd_j +e_5, \end{aligned}$$

where $d_j$ is the regression coefficient for mediator $M_{j}$ and $e_5$ is the residual. $R^2_{Mediated}$ can be interpreted as that in commonality analysis [12]: the variance that is common to both the independent variable and the mediator(s), which is evaluated by the difference in the variance of the dependent variable that is explained by the exposure ($r^2_{Y,X}$) and the additional variance that can be explained by the exposure after taking into account the mediators ($R^2_{Y,MX}-R^2_{Y,M}$), i.e., represented by equation (5). $R^2_{mediated}$ does not directly sum up the $a_jb_j$ from individual pathways with different directions, avoiding the aforementioned problems of the first-moment measures. Recently, the $\nu$ measure, a variant of the R$^2$ measure [32], was extended to multiple-mediator models in the structural equation modeling framework. In fact, under our assumption of undirected correlation among *M*, the extended $\nu$ measure is reduced to $(\sum _{j=1}^p a_jb_j)^2$, i.e., the squared product measure. Therefore, $\nu$ was modified to be a first-moment measure in this case, losing benefits of a second-moment measure.

A major concern of using the R$^2$ measure under a single-mediator model is that it has a negative value in some situations. We discuss this matter thoroughly in Additional file 1: Section 1.2 by showcasing that $R^2_{Mediated}$ can be negative as a difference-in-R$^2$ measure, although it may not happen frequently under a high-dimensional setting. Moreover, we have established several additional appealing properties for the R$^2$-based second-moment measure, including (1) invariance to certain transformations, such as principal component analysis (Additional file 1: Section 1.2.4 Proposition 6), (2) adaptability to a complex dependent structure (Additional file 1: Section 1.3), and (3) robustness to the inclusion of certain types of non-mediators (Additional file 1: Section 1.2.4, Proposition 4).

Another closely related measure is the shared over simple effect (SOS) [33] measure, which is defined as $\text{ SOS }=R^2_{Mediated}/R^2_{Y,X}.$ SOS is a relative measure of $R^2_{Mediated}$. It is the standardized exposure-related variance in the outcome that is shared with the mediator. The relationships among the R$^2$, SOS, product, proportion, and ratio measures are described in Additional file 1: Section 1.2.2. Interestingly, we find that SOS is closely related to the proportion measure, although they have different interpretations: SOS monotonically increases with the absolute value of proportion mediated; on the other hand, it is able to capture some bi-directional mediation effects when the proportion measure cannot.

#### Modelling and estimation

In order to obtain stable estimation under high-dimensional settings, we use the mixed-effect model for improved statistical efficiency, as shown later in the numerical examples. Specifically, we assume that the coefficients for the mediators in models (2) and (6) are random effects. In model (2), $b_j$ is assumed to follow a normal distribution $b_j \sim N(0,\tau _1)$ for $j=1,2,\ldots ,p$ and $e_2 \sim N(0,\phi _1),$ thus

7 $$\begin{aligned} R^2_{Y,MX} =1-\phi _1. \end{aligned}$$

$R^2_{Y,MX}$ can be interpreted as one minus the variance that is unexplained by the independent variable and mediators. Similarly, in model (6), we assume $d_j \sim N(0,\tau _2)$ for $j=1,2,\ldots ,p$ and $e_4 \sim N(0,\phi _2)$, such that $R^2_{Y,M} =1-\phi _2.$

We estimate $\tau _1$,$\tau _2$, $\phi _1$ and $\phi _2$ by the restricted maximum likelihood method, which is consistent under mild conditions [34]. Note that we avoid the direct use of the estimation of a total of 2*p* coefficients ($\beta _1,\ldots , \beta _p, d_1,\ldots , d_p$); instead, we use two parameters ($\phi _1$ and $\phi _2$) to calculate $R^2_{Mediated}$. The estimation of latter is robust to the misspecification of the distribution of the random effects; it has been supported by multiple theoretical studies and real-data analysis [35–37]. Finally, ${\hat{r}}^2_{Y,X} = \sum _{i=1}^n {\hat{y}}_i^2/(n-2),$ where ${\hat{y}}_i$ is the fitted value estimated by MLE in model (1).

When $p<< n$, it is also feasible to estimate the three R$^2$ components by MLE in the fixed-effect models (also proposed in Lachowicz 2018 [38]), and we evaluate its performance in the simulation study for comparison.

#### Mediator variable selection

In the traditional mediation analysis, the mediating variables are hypothesized and selected based on specific research questions and subject matter knowledge. However, hypothesizing and identifying the true mediators becomes much harder in the high-dimensional settings where the bias for estimating the total mediation effects can be induced by failing to identify the true mediators. Inspired by Baron and Kenny 1986 [39], we differentiated the problem into three categories. The first category is the scenario in which the variables falsely identified as mediators are not associated with the exposure, and thus, not in the pathway from the exposure to the outcome (Fig. 2C). For example, some genes influencing lung function are not in the pathway between chronological age and lung function but others, such as a pathway between smoking and lung function. We denote the set of such variables as $$\mathbf {M}^{\mathbf {(1)}}=\{M_j: b_j \ne 0, a_j=0\}$$. Additional file 1: Section 1.2.4, Proposition 4, shows that inclusion of $$\mathbf {M}^{\mathbf {(1)}}$$ provides consistent estimation of $$R^2_{Mediated}$$. The second category is the scenario in which the variables are associated with the exposure, but not the outcome after adjusting for the exposure (Fig. 2D). For example, collagen synthesis is age-related, but genes associated with collagen synthesis may not influence BP. We denote the set of such variables as $$\mathbf {M^{(2)}}=\{M_j:a_j \ne 0, b_j=0\}$$. The inclusion of $$\mathbf {M^{(2)}}$$ could lead to non-zero estimates of the $$R^2_{Mediated}$$ when there is in fact no mediation effect. We further show that the $$R^2_{Mediated}$$ estimate is biased and inconsistent when $$\mathbf {M^{(2)}}$$ are included as mediators in Additional file 1: Section 1.2.4, Proposition 5, as well as the simulation study. Mathematically, the bias comes from $${\hat{R}}^2_{Y,M}$$, where part of the variance of X is falsely added due to the inclusion of $$\mathbf {M}^{\mathbf {(2)}}$$. The third category is the scenario in which noise variables ($$b = 0$$ and $$ a = 0$$) are included, for example, genes not associated with age or the health trait of interest. The inclusion of noise variables does not influence the point estimation of $$R^2_{Mediated}$$ because of the same reason as $$\mathbf {M}^{\mathbf {(1)}}$$. In the steps recommended for mediation analysis [39], $$\mathbf {M}^{\mathbf {(1)}}$$, $$\mathbf {M}^{\mathbf {(2)}}$$, and noise variables are not considered as mediators, and thus should be excluded from mediation analysis. One promising feature of our $$R^2_{Mediated}$$ under high-dimensional settings is its robustness to the inclusion of $$\mathbf {M}^{\mathbf {(1)}}$$ and noise variables. However, the inclusion of $$\mathbf {M}^{\mathbf {(2)}}$$ is clearly problematic, which we use a variable selection method to filter out in model (2) before estimating the $$R^2_{Mediated}$$. For illustration purposes, we denote the true mediators as $$\mathbf{M} $$, the putative mediating variables in the initial assessment as $$\mathbf{M} _0$$, and the variables included in the final mediation model as $${\hat{\mathbf {M}}}$$ in the following context.

**Sure independence screening (SIS)**

To make the high-dimensional problem solvable, we assume that the true mediators are sparse in our motivating question. We adopt iterative SIS, an extension of SIS, to exclude putative mediators with zero coefficients $b_j$’s based on model (2), i.e., the $\mathbf {M}^{\mathbf {(2)}}$ and noise variables. Fan and Lv [16] introduced SIS in the context of ultrahigh-dimensional linear models, which has a sure screening property, i.e., with probability tending to 1, the independence screening technique retains all of the important predictors in the model under certain conditions. The iterative SIS uses marginal and conditional correlations to reduce the dimensionality from high to a moderate scale, for example, $\lfloor n/log(n) \rfloor$, and then additional variable selection via, e.g., minimax concave penalty (MCP), can be improved on both speed and accuracy. The SIS was used in high-dimensional mediation analysis with a focus on hypothesis testing by [40] and later used for variable selection in high-dimensional mediation survival model [41]. For our purposes, we use iterative SIS to handle cases where the regularity conditions of SIS fail due to the existence of $\mathbf{M} ^{(2)}$. For example, some genes maybe jointly uncorrelated with the health trait, but have higher marginal correlations with the trait than true mediators. To obtain valid post-selection inference, we split the data into two halves, using one half to select the true mediator(s) and the other half to estimate $R^2_{Mediated}$ [25, 42]. We establish the consistency of this mixed-model approach to $R^2_{Mediated}$ estimation coupled with iterative SIS-MCP in Additional file 1: Section 1.2.5, i.e., as $n \overset{}{\rightarrow } \infty ,$ ${\hat{R}}^2_{Mediated}(n) \overset{p}{\rightarrow } R^2_{Mediated}.$

**Controlling false discovery rate (FDR)**

Another common practice for filtering out the undesirable variables is to test the marginal association of each potential mediator with *Y* based on the FDR control [20]. We calculated the FDR-adjusted p-values for the $a_j$’s in model (3) and the $b_j^\prime$’s from the models $E(Y) = b_j^\prime M_j + r_j X$, for $j=1,...,p$. When the mediators are conditionally independent given *X*, testing for $b_j^\prime$ is equivalent to testing for $b_j$ in model (2). If either FDR-adjusted p-value of $a_j$ or $b_j$ is larger than 0.1, the variable is excluded from the analysis.

#### Estimating procedure and confidence interval

We describe the estimating procedure incorporating the variable selection step for $R^2_{Mediated}$ in Additional file 1: Section 1.4. It also includes the nonparametric bootstrap method to calculate the percentile CI and a method to adjust for covariates in the mediation models.

### Simulation study

We conducted extensive simulations to evaluate different types of total mediation effect measures, different variable selection methods, and finite-sample performance of the proposed estimating procedure. In Simulation setting I, we compared the bias and variance among the proposed $R^2_{Mediated}$ measure, product, proportion, and ratio measures under both low and high-dimensional settings. Then, we evaluated the variable selection methods regarding the true and false positive rates and the corresponding bias in $R^2_{Mediated}$ (Simulation setting II). Lastly, we reported the finite-sample performance of the consistency of $R^2_{Mediated}$ and the coverage probability of the bootstrap-based confidence interval under different sample sizes in simulation setting III. In general, data were simulated using the same set of coefficients across 500 replications and the true values of $R^2_{Mediated}$ were obtained through Equation (S4) in the Additional file 1. We used the the mixed-effect models to estimate $R^2_{Mediated}$ in all simulation settings and the fixed-effect models for estimation under low-dimensional setting I.

#### Simulation setting I: bias and variance

We evaluated the bias and variance of different types of total mediation effect measures under both low- (L1–L6) and high-dimensional (H1–H12) settings. We are interested in the performance of our proposed measure $R^2_{Mediated}$ when mediation effects are in the same (L5, H5) or different (L1–L4, L6, H1–H4, H6–H12) directions and when three types of previously defined non-mediators are included (L2–L4, H2–H4, H7–H9). In addition, we evaluated its performance when mediators were conditionally dependent in the low-dimensional setting (L6) and when the random effects followed a non-Gaussian distribution under the high-dimensional setting (H6–H12). The simulation set-ups and results for the low-dimensional settings (L1–L6) are included in Additional file 1: Section 1.5.1. For high-dimensional settings, data were generated using model (2) and (3). We set $n=1500$, $e_2 \sim N(0,1), X \sim N(0,1)$, and $r=1$. There were $p_0$ variables in $\mathbf {{M}}_{\mathbf {0}}$, and $\xi =(\xi _1,\xi _2,\ldots ,\xi _{p_0}) \sim N(0,\mathbf{D} _{p_0 \times p_0})$, where $\mathbf{D} _{p_0 \times p_0}$ is the identity matrix. The number of true mediators is *p*.

- (H1) All variables included were true mediators (${\hat{\mathbf {M}}} = \mathbf{M}$, $p_0=p=150$) with different directions: $a_j \sim N(0,0.2)$, $b_j \sim N(0,0.2)$ for $j=1,\ldots ,150$;
- (H2) Adding additional 1350 $$\mathbf{M} ^{(2)}$$ to (H1), i.e., $$p_0=1500$$: $$a_j \sim N(0,0.2)$$, $$b_j =0 $$ for $$j=151,\ldots ,1500$$;
- (H3) Adding additional 1350 $\mathbf{M} ^{(1)}$ to (H1): $a_j = 0$, $b_j \sim N(0,0.2)$ for $j=151,\ldots ,1500$;
- (H4) Adding additional 1350 noise variables to (H1): $a_j = 0$, $b_j =0$ for $j=151,\ldots ,1500$;
- (H5) All variables included were mediators with positive directions: $a_j$ and $b_j$ were the absolute values of the coefficients in (H1);
- (H6) - (H10) Same as (H1) to (H5), except that $a_j$’s and $b_j$’s followed a scaled t-distribution with the degree of freedom equal to 1;
- (H11) Same as (H1) except that $b_j \sim Unif(-0.2,0.2)$ for $j=1,\ldots ,150$;
- (H12) Same as (H1) except that $b_j=0.2$ for $j=1,\ldots ,75$, $b_j=-0.2$ for $j=76,\ldots ,150$.

The true values of each measure are provided in Additional file 1: Tables S2 and S3.

#### Simulation setting II: variable selection

The existence of non-mediator $\mathbf{M} ^{(2)}$ could bias the estimation of our proposed measure, thus we evaluated two commonly used variable selection methods (iterative SIS and marginal association tests controlling FDR) by examining their impact on the bias, standard deviation (SD), and mean square of error (MSE) of the estimation of $R^2_{Mediated}$. We set $n=1500$, $r=3$, $e_2 \sim N(0,1)$, and $X \sim N(0,1)$; $\mathbf{D} _{p_0 \times p_0}$ is the identity matrix. We evaluated the variable selection performance by using (V1) and (V2), representing the scenarios of including two types of non-mediators $\mathbf{M} ^{(1)}$ and $\mathbf{M} ^{(2)}$ with the total number of putative mediators $p_0=1500$; then we increased $p_0$ to 15,000 in (V3) and (V4) to mimic the omics-data application:

- (V1) There were *p* true mediators, and the additional 1350 were $\mathbf{M} ^{(2)}$: $a_j \sim N(0,0.2)$ for $j=1,\ldots ,1500$, and $b_j \sim N(0,0.2)$ for $j=1,\ldots ,p$, $b_j=0$ for $j=p+1,\ldots ,1500$;
- (V2) There were *p* true mediators, and the additional 1350 were $\mathbf{M} ^{(1)}$: $b_j \sim N(0,0.2)$ for $j=1,\ldots ,1500$, and $a_j \sim N(0,0.2)$ for $j=1,\ldots ,p$, $a_j=0$ for $j=p+1,\ldots ,1500$;
- (V3) Adding 13,500 noise variables to (V1): $a_j=b_j=0$ for $j=1501,\ldots ,15{,}000$;
- (V4) There were 1500 $\mathbf{M} ^{(2)}$ and 13,500 noise variables: $a_j \sim N(0,0.2)$ for $j=1,\ldots ,1500$, $a_j=0$ for $j=1505,\ldots ,15{,}000$, and $b_j=0$ for $j=1,\ldots ,15{,}000$.

We varied *p* at 0, 15, 75, 150, and 300, corresponding to 0, 1, 5, 10, and 20 percent of the true mediators in (V1) and (V2). The variable selection was performed in the first half of the data, and the estimation of $R^2_{Mediated}$ was in the second half. The $R^2_{Mediated}$ without variable selection (${\hat{\mathbf {M}}}= \mathbf{M} _0$) and the Lasso regression-based product measure were estimated based on all data, serving as benchmarks.

#### Simulation setting III: consistency, coverage probability, and highly correlated mediators

We further evaluated the following high-dimensional settings: (1) the performance of consistency under finite-sample size $n=750, 1500$, and 3000 with the initial size of ${\mathbf {M}}_{{0}}$ as $p_0=1500$ under four scenarios with different types of non-mediators; (2) coverage probability of the proposed bootstrap-based confidence interval with varying number of true mediators at $p=0,15,150,$ and 300, and sample size at 1500; (3) the finite-sample performance of consistency with highly correlated putative mediators in three additional settings with $p_0=1500$; and (4) the performance of variable selection in the presence of a covariate. The details of the simulation settings were described in Additional file 1: Section 1.7.

## Supplementary Information

**Additional file 1.** More explanation, interpretation, discussion of the proposed measure; additional simulation studies and results; extended real-data application results are provided.

## Data Availability

The transcriptomics data of the FHS study are accessible from the National Center for Biotechnology Information dbGap (https://www.ncbi.nlm.nih.gov/gap/) with access numbers phs000363.v19.p13. The core R code for implementing the proposed method is developed as an R package called “RsqMed”, available at https://cran.r-project.org/web/packages/RsqMed/index.html.

## Abbreviations

$$R^2$$: R-squared
GE: Gene expression
BP: Blood pressure
CI: Confidence interval
FHS: Framingham Heart Study
FDR: False discovery rate
MSE: Mean square error
SD: Standard deviation
FVC: Force vital capacity
SIS: Sure independence screening
SOS: Shared over simple effect
